# Supplementary material for: A Large Language Model–Powered Map of Metabolomics Research
Source: bioRxiv. 2025 Mar 19:2025.03.18.643696. Preprint. [Version 1] doi: 10.1101/2025.03.18.643696 (PMC11957067; doi:10.1101/2025.03.18.643696)
Supplement: 1 [file NIHPP2025.03.18.643696V1-supplement-1.pdf]

bioRxiv preprint doi: https://doi.org/10.1101/2025.03.18.643696; this version posted March 19, 2025. The copyright holder for this preprint (which was not certified by peer review) is the author/funder, who has granted bioRxiv a license to display the preprint in perpetuity. It is made available under aCC-BY 4.0 International license.

SUPPLEMENTARY MATERIALS

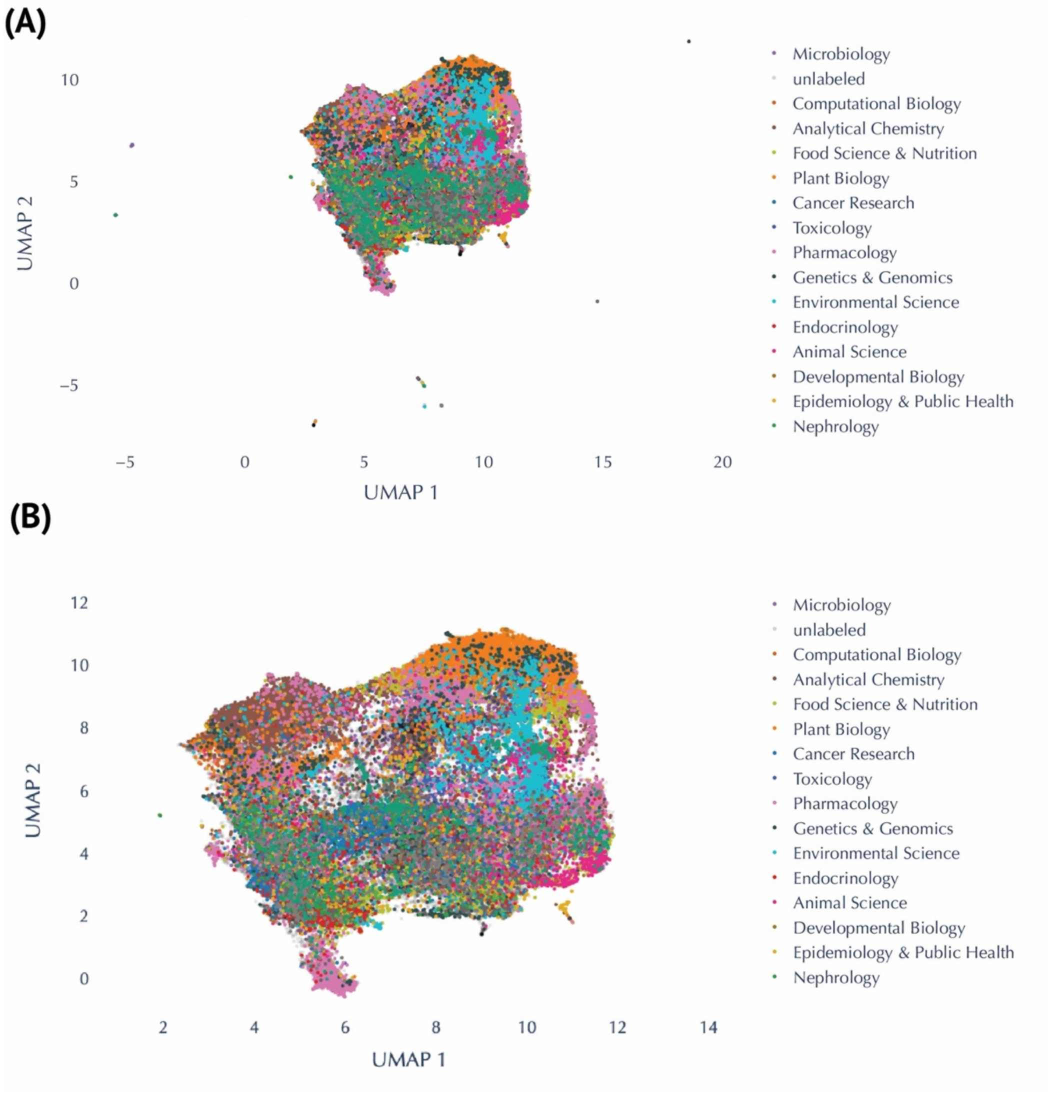

bioRxiv preprint doi: https://doi.org/10.1101/2025.03.18.643696; this version posted March 19, 2025. The copyright holder for this preprint (which was not certified by peer review) is the author/funder, who has granted bioRxiv a license to display the preprint in perpetuity. It is made available under aCC-BY 4.0 International license.

Figure S1. Global visualization of metabolomics research fields using UMAP embeddings.
(A) Two dimensional UMAP projection of 80,656 publications.
(B) A magnified view of two dimensional UMAP projection of 80,656 publications.

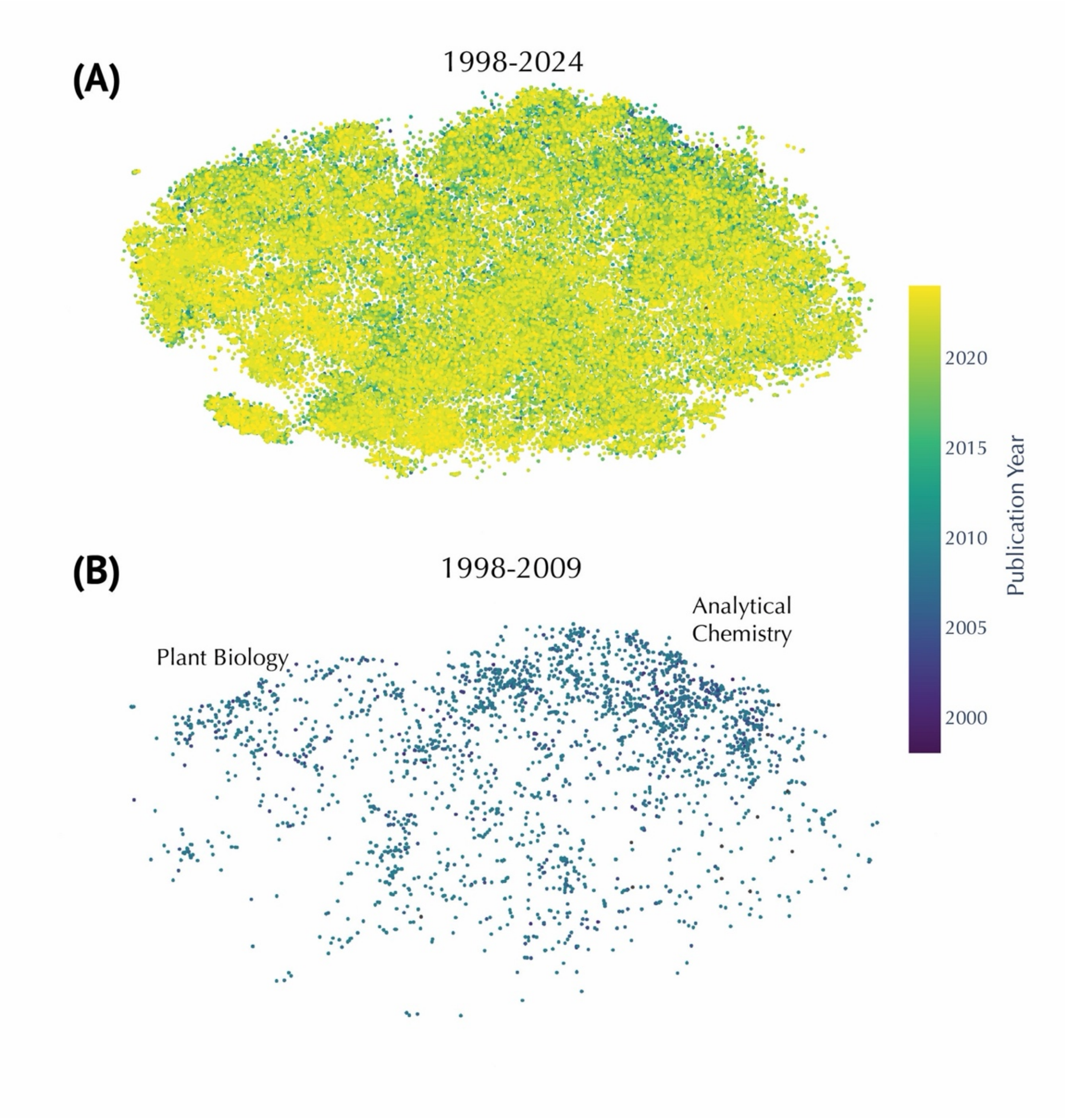

Figure S2. Temporal distribution of metabolomics publications visualized using t-SNE embeddings.
(A) Showing all publications from 1998 to early 2024.
(B) Showing all publications between 1998 and 2009. The publications are concentrated in the ‘Analytical Chemistry’ and ‘Plant Biology’ area of the map.

bioRxiv preprint doi: https://doi.org/10.1101/2025.03.18.643696; this version posted March 19, 2025. The copyright holder for this preprint (which was not certified by peer review) is the author/funder, who has granted bioRxiv a license to display the preprint in perpetuity. It is made available under aCC-BY 4.0 International license.

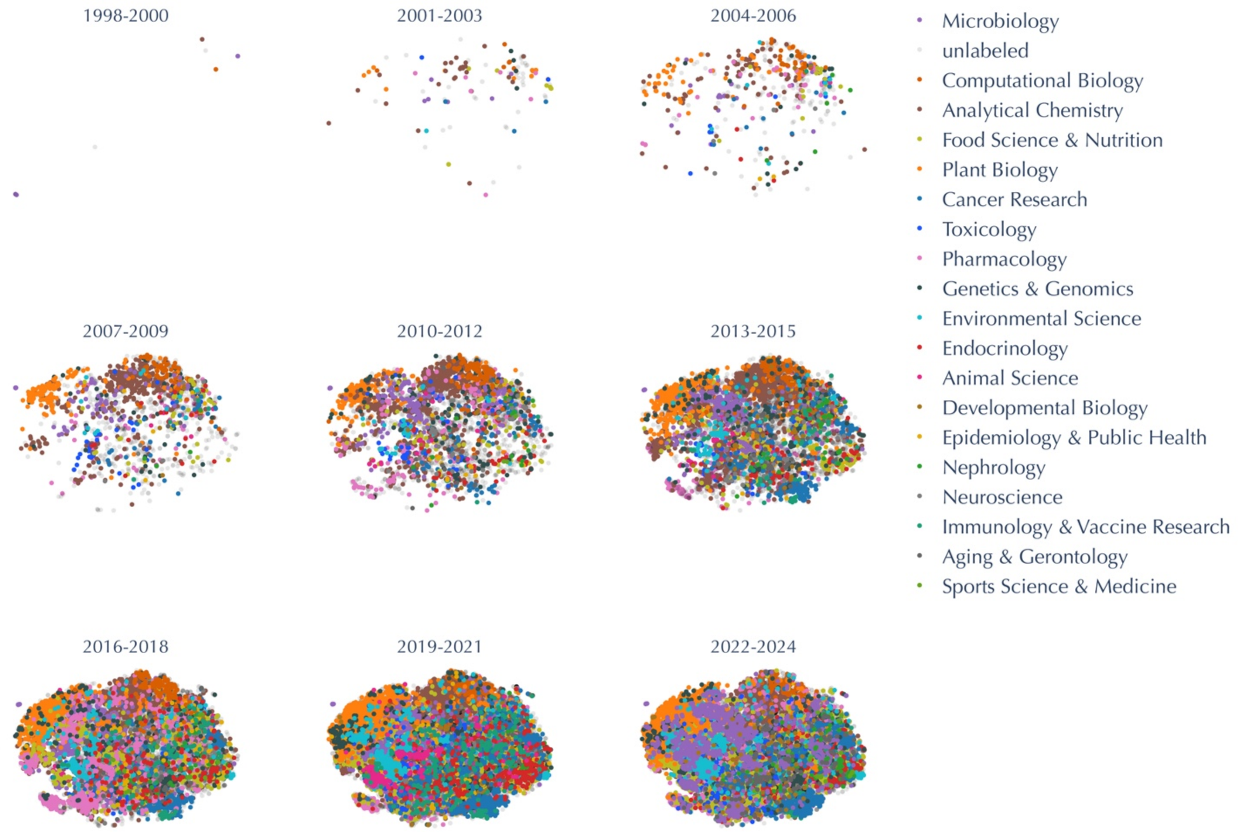

Figure S3: Time-segmented t-SNE visualizations of metabolomics research fields. Clusters represent metabolomics publications divided into eight time periods (1998–2000, 2001–2003, 2004–2006, 2007–2009, 2010–2012, 2013–2015, 2016–2018, 2019–2021, and 2022–early 2024). Each point represents a publication, color-coded by its research domain.

bioRxiv preprint doi: https://doi.org/10.1101/2025.03.18.643696; this version posted March 19, 2025. The copyright holder for this preprint (which was not certified by peer review) is the author/funder, who has granted bioRxiv a license to display the preprint in perpetuity. It is made available under aCC-BY 4.0 International license.

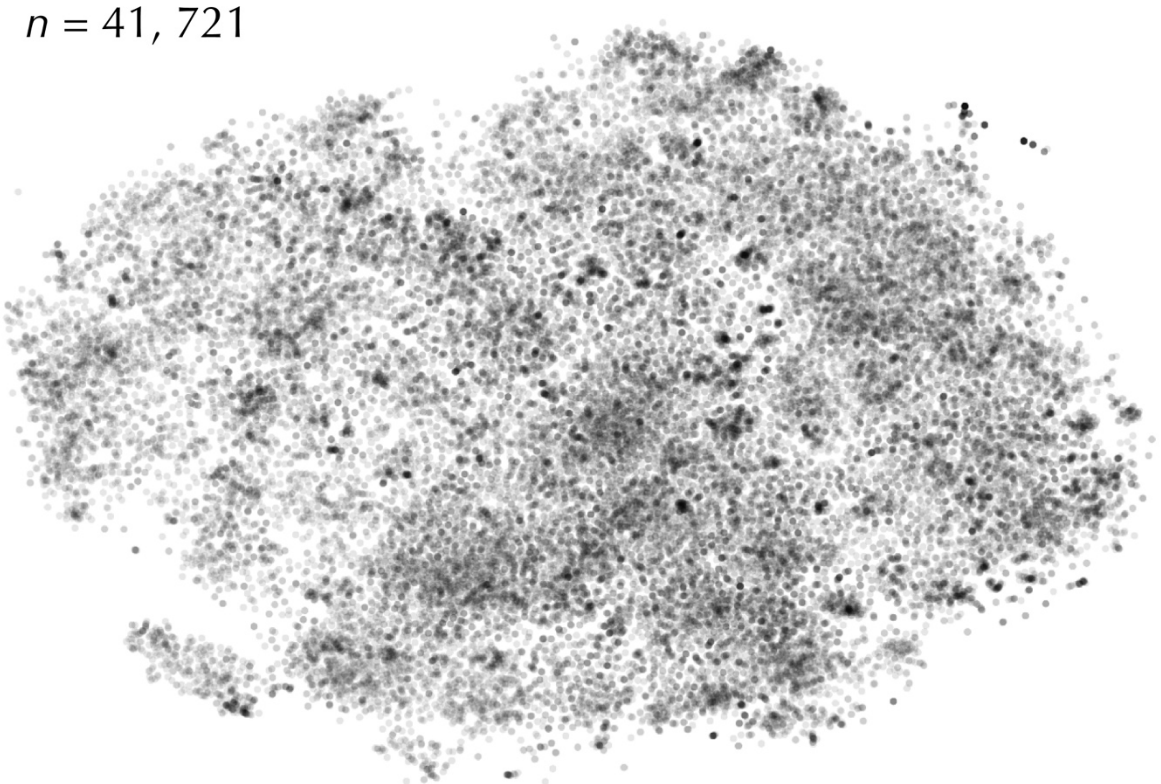

Figure S4: **Unlabeled Cluster of Metabolomics Publications.**
Scatterplot of 41,721 “unlabeled” publications in a t-SNE projection, representing articles not assigned to predefined journal-based categories.

bioRxiv preprint doi: https://doi.org/10.1101/2025.03.18.643696; this version posted March 19, 2025. The copyright holder for this preprint (which was not certified by peer review) is the author/funder, who has granted bioRxiv a license to display the preprint in perpetuity. It is made available under aCC-BY 4.0 International license.

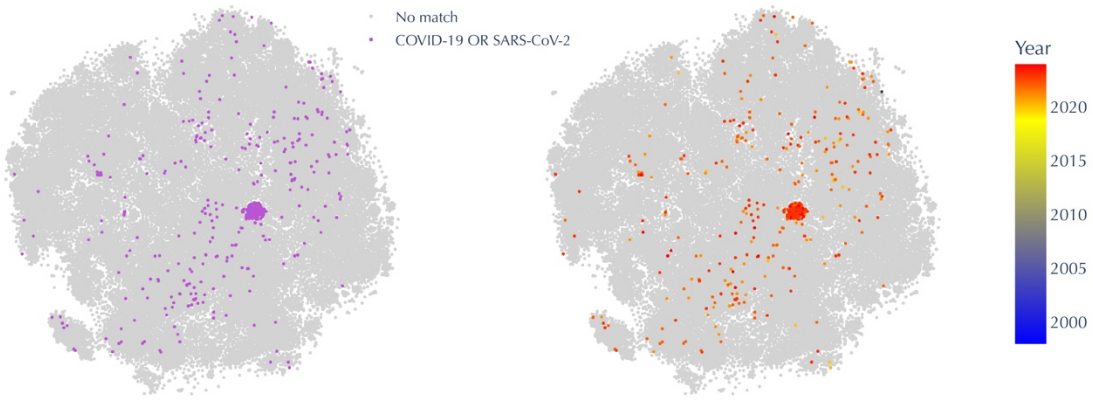

Figure S5: **Embeddings illustrating the impact of COVID-19–related research**. **Left:** Points in **purple** indicate abstracts mentioning “COVID-19 or SARS-CoV-2,” while **gray** points show no mention. **Right:** The same embedding colored by publication year, transitioning from **blue** (earlier) to **red** (more recent). The visible cluster of purple/red points emphasizes the surge of COVID-19 metabolomics studies in recent years.

bioRxiv preprint doi: https://doi.org/10.1101/2025.03.18.643696; this version posted March 19, 2025. The copyright holder for this preprint (which was not certified by peer review) is the author/funder, who has granted bioRxiv a license to display the preprint in perpetuity. It is made available under aCC-BY 4.0 International license.

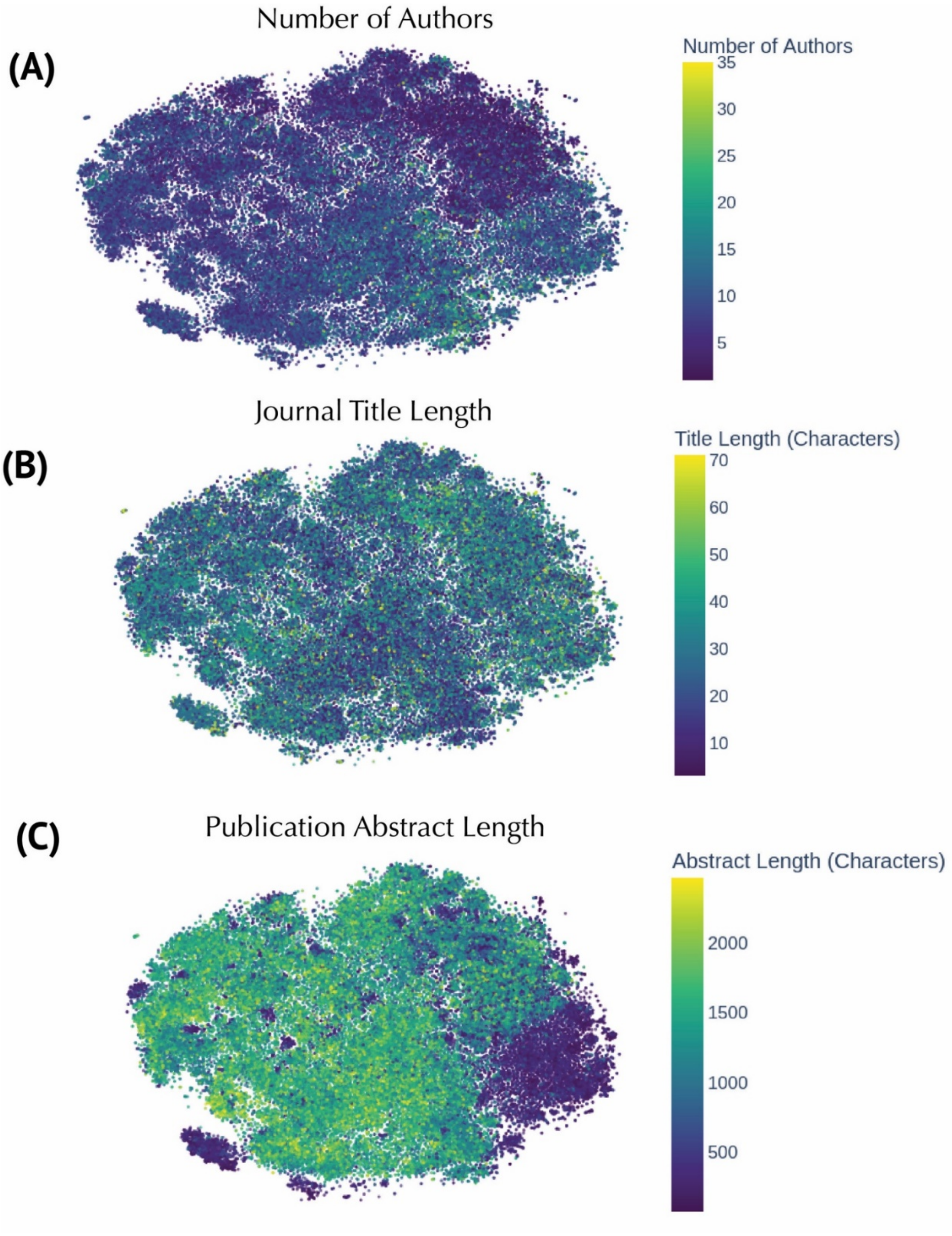

Figure S6: Embeddings colored by publication metadata features.

(A) t-SNE embeddings of metabolomics publications colored by the number of authors per publication, highlighting collaboration trends across the corpus.

(B) Embeddings colored by the length of journal titles.

(C) Embeddings colored by abstract length.

bioRxiv preprint doi: https://doi.org/10.1101/2025.03.18.643696; this version posted March 19, 2025. The copyright holder for this preprint (which was not certified by peer review) is the author/funder, who has granted bioRxiv a license to display the preprint in perpetuity. It is made available under aCC-BY 4.0 International license.

Table S1: Derivative Analysis of Publication Trends in Metabolomics Research. Yearly derivatives were computed to identify periods of growth acceleration and deceleration. The lowest derivatives occurred during the nascent stages of the field (1998–2001), while the highest derivatives were observed during recent years (2019–2022).

Year 1	Year 2	Derivative
	Lowest derivatives	
1998	1999	0
1998	2000	3.00
1998	2001	3.67
2000	2001	5.00
1999	2001	5.50
	Highest derivatives	
2020	2021	1872.00
2019	2021	1653.50
2020	2022	1559.50
2018	2021	1549.33
2019	2022	1518.00

Table S2: Years with Consistently High and Low Rates of Publication Change in Metabolomics Research. Mean and standard deviation (Std) rates of change were calculated for each starting year (Year1). High-growth years (e.g., 2011–2021) were characterized by substantial adoption of metabolomics techniques, while low-growth years (e.g., 1998–2002) reflect the early development of the field. Thresholds for high and low growth were defined as the mean rate of change ± one standard deviation.

Year1	Mean	Std
	Years with consistently high rate of change	
2011	627.57	179.10
2013	766.47	176.62
2014	796.21	214.89
2015	924.82	186.25
2016	942.88	264.33
2017	1151.25	217.46
2018	1412.38	98.406
2019	1477.12	147.45
2020	1563.06	307.18
2021	1098.75	209.66
Year1	Mean	Std
	Years with consistently low rate of change	
1998	159.94	155.85
1999	176.80	161.29
2000	195.68	166.85
2001	218.96	170.63
2002	242.70	175.83

bioRxiv preprint doi: https://doi.org/10.1101/2025.03.18.643696; this version posted March 19, 2025. The copyright holder for this preprint (which was not certified by peer review) is the author/funder, who has granted bioRxiv a license to display the preprint in perpetuity. It is made available under aCC-BY 4.0 International license.

Table S3: **Publication counts by research fields in metabolomics.** Breakdown of all publications across 18 predefined research domains, based on journal titles. The "unlabeled" category accounts for publications without domain classification.

**Category**	**Count**
unlabeled	41721
Analytical Chemistry	8981
Plant Biology	4648
Pharmacology	4229
Food Science & Nutrition	4053
Microbiology	3605
Cancer Research	2657
Environmental Science	1945
Genetics & Genomics	1312
Toxicology	1175
Neuroscience	980
Endocrinology	953
Computational Biology	943
Immunology & Vaccine Research	924
Animal Science	876
Epidemiology & Public Health	547
Aging & Gerontology	448
Developmental Biology	389
Nephrology	277
Sports Science & Medicine	76

bioRxiv preprint doi: https://doi.org/10.1101/2025.03.18.643696; this version posted March 19, 2025. The copyright holder for this preprint (which was not certified by peer review) is the author/funder, who has granted bioRxiv a license to display the preprint in perpetuity. It is made available under aCC-BY 4.0 International license.

Table S4: Publication counts by research fields in metabolomics from 1998 to 2009.

Category	Count
unlabeled	1116
Analytical Chemistry	577
Plant Biology	205
Pharmacology	155
Microbiology	109
Computational Biology	106
Genetics & Genomics	82
Food Science & Nutrition	72
Toxicology	61
Cancer Research	45
Endocrinology	21
Environmental Science	20
Neuroscience	18
Nephrology	9
Animal Science	8
Epidemiology & Public Health	7
Developmental Biology	6
Immunology & Vaccine Research	6
Aging & Gerontology	2

bioRxiv preprint doi: https://doi.org/10.1101/2025.03.18.643696; this version posted March 19, 2025. The copyright holder for this preprint (which was not certified by peer review) is the author/funder, who has granted bioRxiv a license to display the preprint in perpetuity. It is made available under aCC-BY 4.0 International license.

Table S5. Publication counts by topics in metabolomics.
Breakdown of all publications across the topics generated by GPT4o mini and the BERTopic pipeline. The "outlier" category accounts for publications without topic assignment.

Custom Name	Count
Outlier	14980
Plant Stress Response Mechanisms	19258
Metabolic Profiles and Dysregulation	9730
Cancer Metabolism and Therapy Resistance	7254
Metabolomics Data Analysis and Integration	6499
Gut Microbiota and Metabolomic Interactions	4313
Metabolomics in Neurodegenerative Disorders	2723
Environmental Toxicology and Metabolism	2389
Metabolomics in Animal Nutrition	1971
Microbiota-Gut-Brain Axis Interactions	1661
Kidney Disease Metabolomics and Biomarkers	1479
Metabolomics in Rheumatoid Arthritis	1318
Maternal Metabolomic Changes in Pregnancy	1238
Lung Disease and Metabolic Dynamics	1186
NMR Spectroscopy Innovations in Metabolomics	779
COVID-19 Metabolomic and Immune Responses	776
Lipid Profiling Techniques	687
Host-Parasite Metabolic Interactions	656
Male Fertility and Reproductive Metabolomics	652
Ocular Metabolomics and Disease Mechanisms	604
Salivary Metabolomics in Oral Health	503

bioRxiv preprint doi: https://doi.org/10.1101/2025.03.18.643696; this version posted March 19, 2025. The copyright holder for this preprint (which was not certified by peer review) is the author/funder, who has granted bioRxiv a license to display the preprint in perpetuity. It is made available under aCC-BY 4.0 International license.

Appendix A: Code Showing Label Mapping for Label Inference using Journal Titles

```
# Define candidate labels
candidate_labels = [
    "Oncology + Cancers + Cancer + Oncogene + Anticancer + Oncotarget + Oncoimmunology + Carcinogenesis +
Metastasis + Tumori + Tumor",
    "Plant + Botany + Planta + Phytopathology + Horticulture",
    "Kidney + Nephrology + Nephron + Dialysis",
    "Endocrinology + Endocrine + Hormone + Endocrinological",
    "Microbiology + Bacteriology + Leeuwenhoek + Yeast + mBio + mSphere + Microbiome + Microbes +
MicrobiologyOpen + mSystems + Microorganisms",
    "Analytical Chemistry + Chromatography + Mass Spectrometry + Analyst + Analytica + Bioanalysis +
Separation + Spectroscopy",
    "Pharmacology + Pharmaceutical + Pharmacological + Pharmacologica + Pharmacogenomics +
Pharmacogenetics + Drug + Drugs + Ethnopharmacology + Medicinal + Natural + Pharmaceutics +
Phytopharmacology + Pharmacognosy",
    "Neuroscience + Neurochemistry + Brain + Neuroinflammation + Neurology + Neurochemical +
Neuroimmunology + Cerebral + Neuroimage + Neurotrauma + Neurological + Neuro-oncology + Neurodegeneration
+ Neuropsychiatric + Neuropsychiatry + Neuroendocrinology + Headache",
    "Nutrition + Food + Foods + Nutritional + Nutrients + Dairy + Foodborne",
    "Toxicology + Toxicological",
    "Environmental + Environment + Hazardous + Pollution",
    "Animal + Animals + Poultry + Veterinary + Livestock + Ruminant + Theriogenology + Zoology",
    "Sports + Sport + Exercise + Knee + Arthroscopy + Athletic",
    "Epidemiology + Infectious + Public",
    "Developmental + Development",
    "Gerontology + Ageing + Geriatrics + Aging + Geroscience",
    "Immunology + Immunity + Leukocyte + Autoimmunity + Immunobiology + Immunotargets + Immunotherapy +
Vaccines",
    "Bioinformatics + Chemometrics + Cheminformatics + Computational",
    "Genetics + Genomics + Genome"
]

# The label mapping
label_mapping = {
    "Oncology + Cancers + Cancer + Oncogene + Anticancer + Oncotarget + Oncoimmunology + Carcinogenesis +
Metastasis + Tumori + Tumor": "Cancer Research",
    "Plant + Botany + Planta + Phytopathology + Horticulture": "Plant Biology",
    "Kidney + Nephrology + Nephron + Dialysis": "Nephrology",
    "Endocrinology + Endocrine + Hormone + Endocrinological": "Endocrinology",
    "Microbiology + Bacteriology + Leeuwenhoek + Yeast + mBio + mSphere + Microbiome + Microbes +
MicrobiologyOpen + mSystems + Microorganisms": "Microbiology",
    "Analytical Chemistry + Chromatography + Mass Spectrometry + Analyst + Analytica + Bioanalysis +
Separation + Spectroscopy": "Analytical Chemistry",
    "Pharmacology + Pharmaceutical + Pharmacological + Pharmacologica + Pharmacogenomics +
Pharmacogenetics + Drug + Drugs + Ethnopharmacology + Medicinal + Natural + Pharmaceutics +
Phytopharmacology + Pharmacognosy": "Pharmacology",
    "Neuroscience + Neurochemistry + Brain + Neuroinflammation + Neurology + Neurochemical +
Neuroimmunology + Cerebral + Neuroimage + Neurotrauma + Neurological + Neuro-oncology + Neurodegeneration
+ Neuropsychiatric + Neuropsychiatry + Neuroendocrinology + Headache": "Neuroscience",
    "Nutrition + Food + Foods + Nutritional + Nutrients + Dairy + Foodborne": "Food Science & Nutrition",
    "Toxicology + Toxicological": "Toxicology",
    "Environmental + Environment + Hazardous + Pollution": "Environmental Science",
    "Animal + Animals + Poultry + Veterinary + Livestock + Ruminant + Theriogenology + Zoology": "Animal
Science",
    "Sports + Sport + Exercise + Knee + Arthroscopy + Athletic": "Sports Science & Medicine",
    "Epidemiology + Infectious + Public": "Epidemiology & Public Health",
    "Developmental + Development": "Developmental Biology",
    "Gerontology + Ageing + Geriatrics + Aging + Geroscience": "Aging & Gerontology",
    "Immunology + Immunity + Leukocyte + Autoimmunity + Immunobiology + Immunotargets + Immunotherapy +
Vaccines": "Immunology & Vaccine Research",
    "Bioinformatics + Chemometrics + Cheminformatics + Computational": "Computational Biology",
    "Genetics + Genomics + Genome": "Genetics & Genomics"
}
```

bioRxiv preprint doi: https://doi.org/10.1101/2025.03.18.643696; this version posted March 19, 2025. The copyright holder for this preprint (which was not certified by peer review) is the author/funder, who has granted bioRxiv a license to display the preprint in perpetuity. It is made available under aCC-BY 4.0 International license.

Appendix B: Prompt for Topic Modelling

```
# A prompt consisting of a system prompt, an in-context example, and a main prompt

openai_prompt = """
You are an expert in metabolomics and scientific literature analysis. Your task
is to generate concise, informative topic labels for collections of metabolomics
abstracts from PubMed. Each topic label should be no more than 6 words long and
should capture the essence of the metabolomics research described.

Here is an example:
I have a topic that contains the following metabolomics abstracts:
- This study investigates the metabolic profiling of plasma samples from patients
with type 2 diabetes using LC-MS/MS. We identified several key metabolites
associated with insulin resistance.
- Our research focuses on the application of NMR spectroscopy to analyze urine
samples for early detection of kidney disease. The metabolic signatures
revealed potential biomarkers.
- We employed GC-MS to examine the metabolome of cancer cells under hypoxic
conditions. The results showed significant alterations in glucose and glutamine
metabolism.

The topic is described by the following keywords: 'metabolomics, LC-MS, NMR,
biomarkers, disease detection.'

A suitable topic label would be: Disease Biomarker Discovery.

Now, based on the information provided below, please create a concise topic
label for this metabolomics topic in 6 words or fewer.

Documents: [DOCUMENTS]
Keywords: [KEYWORDS]

Return only the topic label, nothing more. Make sure it is in the following format:
topic: <topic label>
"""
```
